# Efficacy and utility of preemptive anti-Gr(+) coverage in selected critically ill patients

**DOI:** 10.1186/cc12011

**Published:** 2013-03-19

**Authors:** VT Todorova, SM Milanov, G Georgiev, M Milanov

**Affiliations:** 1 Emergency Hospital 'Pirogov', Sofia, Bulgaria

## Introduction

Based on the results of our previous studies [1] we have identified clinical risk factors for the emergence of Gr(+) infections in our ICU and we have developed a new algorithm for combating them. The choice of the particular antibiotic drug is guided by additional risk factors for severity of illness and data on the infectious focus. The response to therapy and its duration are also stated. The aim of the current study was to evaluate the efficacy and safety of this preemptive approach.

## Methods

A randomized prospective controlled trial was carried out from September 2010 to September 2012. Patients were submitted to block randomization and stratified on the basis of their initial SAPS II exp score. Antibiotic therapy was started on the day of inclusion in the treatment group and only with proven Gr(+) pathogen in the control group. Initial data were gathered on demographics, diagnosis, proven risk factors for sepsis-related mortality, severity of inflammatory response, ventilator-associated pneumonia and organ dysfunction. Dynamics of SIRS, CPIS and SOFA scores, subsequent infectious isolates, ventilator-free days, length of ICU stay and outcome were followed for each patient.

## Results

A total of 170 patients were enrolled. No statistically significant differences in their basal characteristics were found. The subsequent score values, length of ICU stay and the number of ventilator-free days were also comparable between groups. The majority of Gr(+) pathogens were isolated between 6 and 10 days of inclusion. No differences were found regarding the concomitant Gr(-) flora and the related antibiotic therapy. The new organ dysfunction severity was similar in both groups (*P *= 0.37). The in-hospital mortality was 26.2% in the treatment group versus 18.6% in the control group (*P *= 0.56). Significant differences between the Kaplan-Meier estimates of survival were also not found (log-rank test *P *= 0.81). No major adverse reactions were observed.

## Conclusion

The implementation of this new policy failed to reduce the degree of organ dysfunction severity and was not associated with significant survival benefit. Moreover, even though it did not reach statistical significance, a second peak of Gr(+) isolates was observed as a possible complication of the preemptive therapy. Whether this approach could lead to vancomycin MIC creep or there could still be a niche for it later in the course of treatment and/or in nontrauma patients remains to be further explored.
